# Ultrarapid detection of SARS-CoV-2 RNA using a reverse transcription–free exponential amplification reaction, RTF-EXPAR

**DOI:** 10.1073/pnas.2100347118

**Published:** 2021-08-16

**Authors:** Jake G. Carter, Lorea Orueta Iturbe, Jean-Louis H. A. Duprey, Ian R. Carter, Craig D. Southern, Marium Rana, Celina M. Whalley, Andrew Bosworth, Andrew D. Beggs, Matthew R. Hicks, James H. R. Tucker, Timothy R. Dafforn

**Affiliations:** ^a^School of Chemistry, University of Birmingham, Birmingham B15 2TT, United Kingdom;; ^b^School of Biosciences, University of Birmingham, Birmingham B15 2TT, United Kingdom;; ^c^Linear Diagnostics Ltd, Birmingham B15 2SQ, United Kingdom;; ^d^C2JF Solutions LLP, Liverpool L24 9LG, United Kingdom;; ^e^Institute of Cancer and Genomics, University of Birmingham, Birmingham B15 2TT, United Kingdom;; ^f^Clinical Virology, Clinical Laboratory Services, University Hospitals Birmingham NHS Foundation Trust, Birmingham B15 2GW, United Kingdom

**Keywords:** RNA detection, COVID-19 assay, nucleic acids, EXPAR, isothermal amplification

## Abstract

A rapid isothermal method for detecting severe acute respiratory syndrome coronavirus 2 (SARS-CoV-2), the virus responsible for COVID-19, is reported. The procedure uses an unprecedented reverse transcription–free (RTF) approach for converting genomic RNA into DNA. This involves the formation of an RNA/DNA heteroduplex whose selective cleavage generates a short DNA trigger strand, which is then rapidly amplified using the exponential amplification reaction (EXPAR). Deploying the RNA-to-DNA conversion and amplification stages of the RTF-EXPAR assay in a single step results in the detection, via a fluorescence read-out, of single figure copy numbers per microliter of SARS-CoV-2 RNA in under 10 min. In direct three-way comparison studies, the assay has been found to be faster than both RT-qPCR and reverse transcription loop-mediated isothermal amplification (RT-LAMP), while being just as sensitive. The assay protocol involves the use of standard laboratory equipment and is readily adaptable for the detection of other RNA-based pathogens.

## Significance

We report a rapid COVID-19 assay that gives a sample-to-signal time of under 10 min. The current gold-standard COVID-19 assay uses PCR, where strands of DNA are copied (amplified) many times to generate a read-out signal. However, as the virus genome is RNA, first conversion into DNA is required using reverse transcription (RT) before amplification. While just as sensitive, our assay is faster because 1) we have designed a method for generating DNA (the trigger strand) from RNA, bypassing the lengthy RT step, and 2) a quicker amplification process than PCR, called exponential amplification reaction (EXPAR), is used to amplify the trigger. This methodology could ultimately be applied to any RNA-based assay, including the detection of other infectious agents.

In order to reduce the rate of spread of COVID-19 an accurate and efficient virus testing strategy is imperative. A key part of this strategy is continuous assay development, with the aim of reducing detection times and increasing sample throughput. The research community and diagnostics industry have responded rapidly to this unprecedented crisis in developing a range of detection platforms (1–6). The most sensitive assays detect viral RNA, with the current gold standard being RT-qPCR, a two-step assay that takes more than 60 min per sample. First, reverse transcriptase converts viral RNA to complementary DNA (cDNA), a process that can take up to 30 min (7). Then, a qPCR amplifies the cDNA, which is detected using a fluorescent dye, a process that takes up to an hour (8–11). To reduce assay times, a plethora of new approaches to severe acute respiratory syndrome coronavirus 2 (SARS-CoV-2) detection have appeared in the literature over the past year (1). As far as nucleic acid amplification tests are concerned, which are more sensitive than current 30-min lateral flow antigen (immunoassay) tests (12, 13), focus has turned toward isothermal DNA amplification approaches, which increase amplification speeds and hence reduce assay times. The most common isothermal amplification system is loop-mediated isothermal amplification (LAMP) (8, 14). RT-LAMP assays have been developed for SARS-CoV-2 but take, on average, 20 min for a result, with further decreases in LAMP assay time proving challenging (5, 6, 15, 16). Herein, we demonstrate an alternative isothermal approach based on the exponential amplification reaction (EXPAR) (17, 18), a simpler and faster amplification method than LAMP. By combining EXPAR with an unprecedented reverse transcription–free (RTF) step to convert RNA into DNA, this assay, RTF-EXPAR, can accurately identify 7.25 copies per µL of SARS-CoV-2 RNA in less than 10 min.

## Results and Discussion

**Fig. 1.**
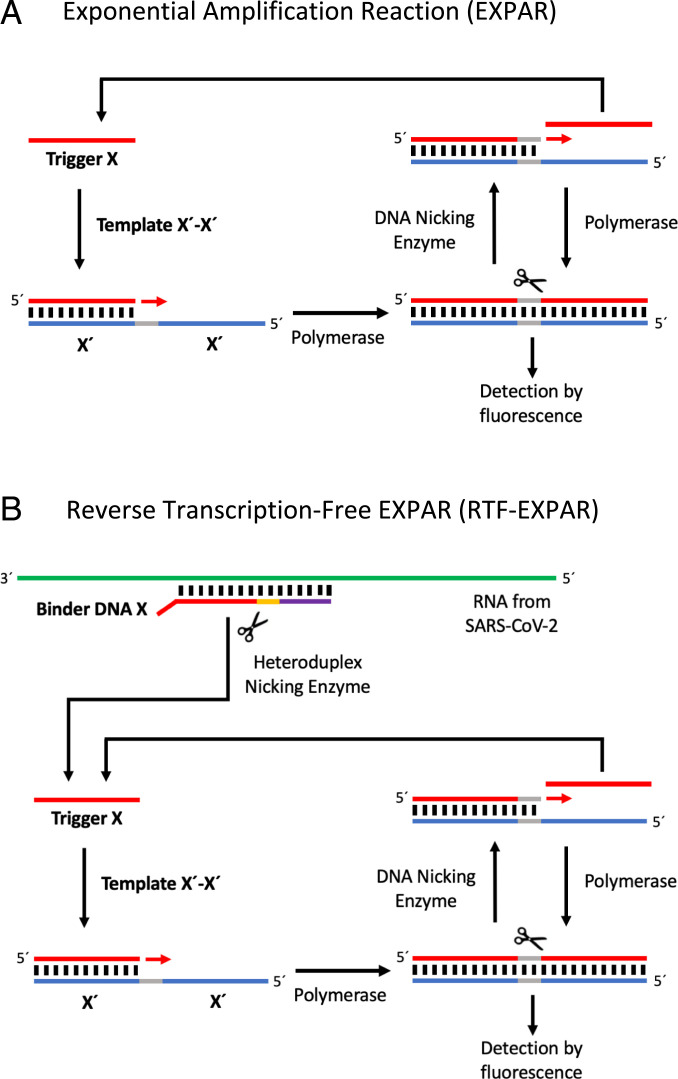
(*A*) Reaction scheme for EXPAR: **Trigger X** anneals to **Template X′-X′** and is extended by a DNA polymerase (*Bst* 2.0 polymerase); the top strand of the newly formed duplex DNA is then cut by a nicking enzyme (Nt.*Bst*NBI); the released DNA (which is displaced by DNA polymerase in a subsequent extension reaction) is identical to **Trigger X** and is therefore able to prime another **Template X′-X′**. (*B*) Reaction scheme for RTF-EXPAR: **Binder DNA** **X** anneals to viral RNA; the DNA strand of the DNA:RNA heteroduplex is cut by the restriction endonuclease *Bst*NI, which acts as a nicking enzyme by cutting the DNA strand only; the released DNA strand is **Trigger X**, which is then amplified by EXPAR.

The key to the speed of EXPAR is twofold; first, the amplification occurs at a single temperature, thus avoiding lengthy heating and cooling steps, and second, the amplicon is relatively small (typically 15 to 20 bases long), compared to both PCR and LAMP. These two factors result in EXPAR, once triggered, producing up to 10^8^ strands of DNA product in a matter of minutes (17–19). A single-stranded DNA fragment (the trigger) starts the EXPAR reaction by binding a DNA template. Large quantities of short double-stranded DNA sequences are then generated in an isothermal cycle involving a DNA polymerase to extend the sequence and a nicking endonuclease to cut it, while leaving the template intact (Fig. 1*A*). As with the RT-qPCR COVID-19 assay, duplex formation is monitored spectroscopically using a fluorescent intercalating dye, e.g. SYBR Green.

A crucial element to developing a successful EXPAR assay is the identification of optimal nucleotide sequences in the target genome. Qian et al. previously found that the type of trigger sequence used in EXPAR plays a vital role in determining its efficiency (17, 20). Using their approach, we designed a 17-mer DNA trigger for EXPAR (**Trigger X**; Fig. 1*A* and Table 1) containing a sequence complementary to one within the conserved gene *Orf1ab* in the SARS-CoV-2 genome (https://www.ncbi.nlm.nih.gov/nuccore/MN908947.3?report=fasta). We first analyzed the speed and sensitivity of EXPAR using **Trigger X** in the presence of **Template X′-X′** (*SI Appendix*, Fig. S1). Rapid rises in SYBR Green fluorescence were observed, with amplification times revealing an expected dependence on trigger concentration (e.g., time to 10-sigma: 3.17 ± 0.14 min at 10 nM and 8.67 ± 1.08 min at 10 pM). These results demonstrate that EXPAR is a faster amplification method than LAMP. Next, we analyzed the specificity of the reaction by investigating three other triggers (**Triggers A**, **B**, and **C**), each at a concentration of 10 nM, that were noncomplementary to **Template X′-X′** (*SI Appendix*, Fig. S2). Each of these three triggers produced no signal within 10 min under the same conditions, confirming the specificity of the EXPAR reaction, with only the trigger sequence fully complementary to the template (**Trigger X**) resulting in rapid amplification.

**Table 1.** Oligonucleotides used in the study

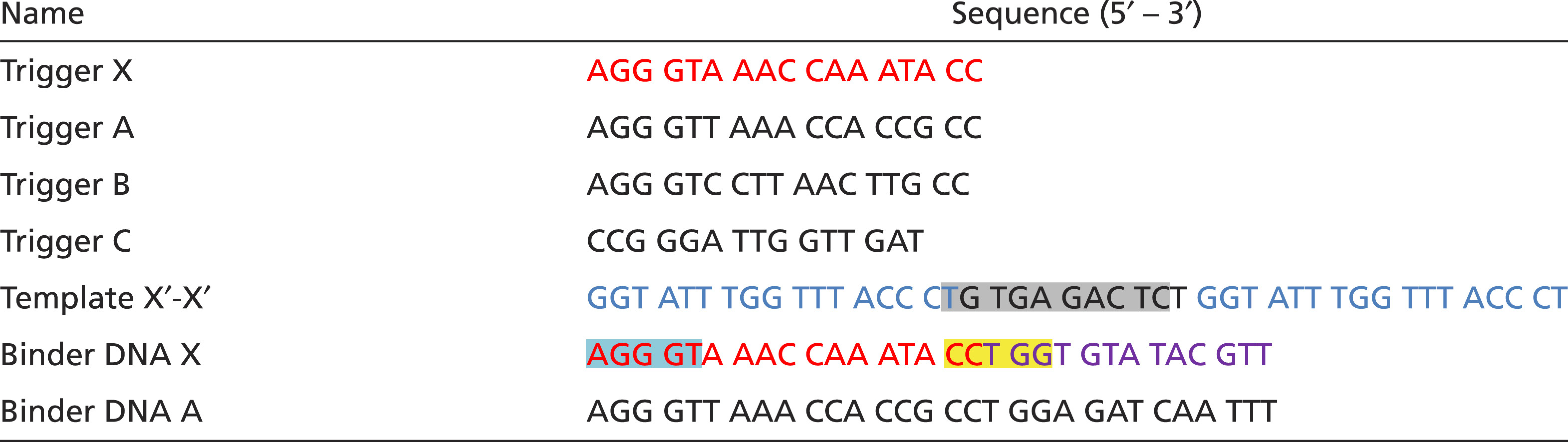

Key: turquoise highlight, non-RNA-binding fragment in **Binder DNA X** sequence; yellow highlight, *Bst*NI recognition site (complementary to required 5′-CCAGG-3′ sequence in RNA target); gray highlight, Nt.*Bst*NBI recognition site; blue letters, two complementary sequences to **Trigger X**; red letters, **Trigger X** sequence in **Binder DNA** **X** sequence; purple letters, 3′ end of **Binder DNA** **X** sequence cleaved by *Bst*NI enzyme.

In the standard RT-qPCR COVID-19 assay, reverse transcriptase converts the RNA of SARS-CoV-2 into cDNA prior to amplification (discussed above). The speed of this initial polymerization reaction is a significant limitation for this or potentially any other RNA detection method that proceeds via cDNA amplification, including LAMP or EXPAR. We hypothesized that a faster method could be achieved by generating a short DNA trigger sequence directly from the RNA genomic strand, without the need for the lengthy reverse transcription step. Murray et al. had previously demonstrated that the restriction enzyme *Bst*NI could act as a nicking enzyme by selectively cleaving DNA within RNA:DNA heteroduplexes (21). We considered that this enzyme could be used to generate the desired DNA fragment for triggering the EXPAR reaction. To achieve this, we designed a 30-mer DNA oligonucleotide (called **Binder DNA X**; Table 1) possessing a 5-base recognition site for *Bst*NI, as well as two partially overlapping sequence stretches complementary to part of *Orf1ab* in the SARS-CoV-2 RNA genome and the EXPAR DNA template (**Template X′-X′**). Site-selective cleavage of **Binder DNA** **X** using *Bst*NI would only occur in the presence of the RNA target from SARS-CoV-2, generating a shorter strand of DNA, **Trigger X** (Fig. 1*B*). This shorter 17-mer strand would now release from the heteroduplex and bind preferably to the DNA template, as it can still form a fully complementary duplex with the latter. Binding to the template would trigger EXPAR, with the newly released RNA strand able to bind more **Binder DNA** **X** to generate more **Trigger X**.

**Fig. 2.**
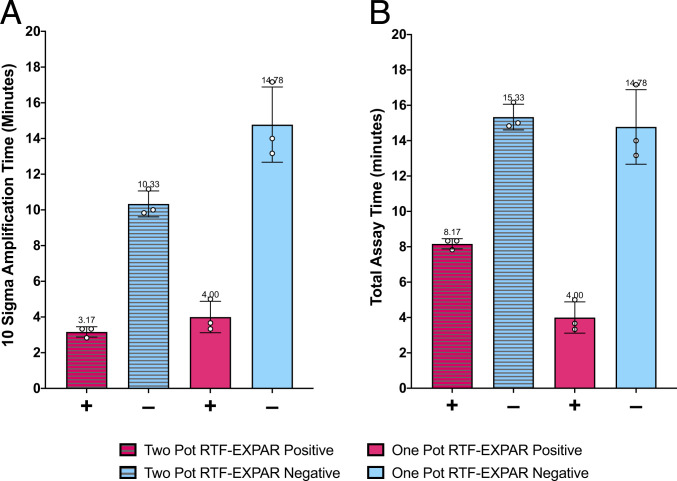
RTF-EXPAR assay data (Protocol 1, Sample Batch 1) for SARS-CoV-2 RNA detection (73 copies per µL, *n* = 3), showing (*A*) the mean time for the amplification reaction only and (*B*) the mean total assay time from RNA sample to signal. Each run time was calculated to be the point at which the fluorescence signal was greater than 10 SDs from the baseline signal (10-sigma time). Error bars in datasets are the SDs of the 10-sigma time. Signals observed for negative samples at >10 min are attributed to amplification arising from nonspecific interactions.

Applying our RTF-EXPAR approach in a two-stage process, we first undertook an enzymatic digestion at 50 °C for 5 min of **Binder DNA** **X** (1 µM) in the presence of a sample of SARS-CoV-2 viral RNA (73 copies per µL) (22) extracted from specimens obtained from Public Health England (PHE), Porton Down (Sample Batch 1, *Materials and Methods*), before adding this solution to the EXPAR reagent mix for the amplification step (Protocol 1). This stage, performed in triplicate, gave an amplification time of 3.17 ± 0.24 min, whereas no amplification was observed for the negative sample within 10 min (Fig. 2 and *SI Appendix*, Fig. S3). To increase the speed of the RTF-EXPAR assay further, we next investigated a “one-pot” approach by introducing *Bst*NI and **Binder DNA** **X** to the EXPAR reagents at the same time, before incubating and amplifying simultaneously at 50 °C. These assay conditions gave an amplification time of only 4.00 ± 0.72 min for the positive sample, halving the total assay time compared to the “two-pot” method (Fig. 2 and *SI Appendix*, Fig. S4). Once again, no signal change for the negative sample was observed within 10 min. As expected, this was also the case for control experiments on a sample of RNA isolated from the CFPAC-1 human ductal pancreatic adenocarcinoma cell line (see *SI Appendix*, Fig. S5) and on the positive RNA sample (73 copies per µL) either in the absence of one of the reagents (*Bst*NI, **Binder DNA X**, or **Template X′-X′)** or in the presence of an alternative binder strand, **Binder DNA A** (see Table 1 and *SI Appendix*, Fig. S6).

**Fig. 3.**
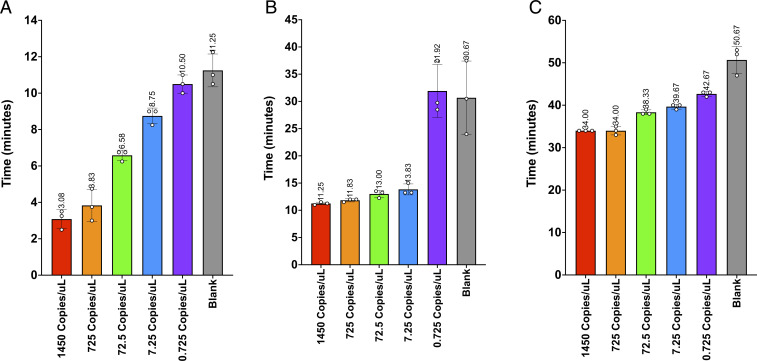
RTF-EXPAR assay data (Protocol 2) for SARS-CoV-2 RNA detection (Sample Batch 2, *n* = 3), showing (*A*) the mean time for the amplification reaction using RTF-EXPAR, (*B*) the mean time for the amplification reaction using RT-LAMP, and (*C*) the mean time for the amplification reaction using RT-qPCR. Each run time was calculated to be the point at which the fluorescence signal was greater than 10 SDs from the baseline signal (10-sigma time). Error bars in datasets are the SDs of the 10-sigma time.

With the “one-pot” RTF-EXPAR conditions established, next we undertook some three-way studies comparing EXPAR with RT-qPCR and RT-LAMP on qPCR instrumentation used for National Health Service (NHS) COVID-19 testing at the University of Birmingham, using a slightly altered protocol (Protocol 2). The first of these was a target dilution study to compare assay sensitivity and speed on viral RNA isolated from specimens supplied by PHE, Porton Down (Sample Batch 2, 1,450 to 0.725 copies per µL; Fig. 3). The “gold-standard” technique RT-qPCR was capable of detecting the lowest RNA concentration of 0.725 copies per µL in under 45 min (42.67 ± 0.47 min, C_T_ = 25) and the highest concentration of 1,450 copies per µL in under 35 min (34.00 ± 0.00 min, C_T_ = 17), with nonspecific amplification (a blank sample containing no RNA) occurring in under 1 h (50.67 ± 2.62 min, C_T_ = 33). As expected, RT-LAMP was found to be quicker than RT-qPCR, demonstrating amplification times of between 11 and 15 min for concentrations ranging from 1,450 copies per µL (11.25 ± 0.20 min) to 7.25 copies per µL (13.83 ± 0.82 min), with the latter being the limit of detection (LOD). Whereas RTF-EXPAR showed a similar sensitivity to RT-LAMP under these conditions, the speed of the amplification reaction was faster still, with 7.25 copies per µL of SARS-CoV-2 RNA detected in under 10 min (8.75 ± 0.35 min) and 1,450 copies per µL detected after just 3.08 ± 0.42 min, a significant improvement over both RT-qPCR and RT-LAMP.

**Fig. 4.**
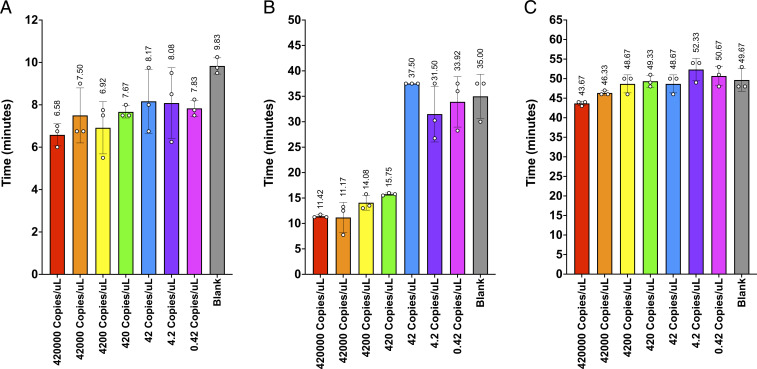
RTF-EXPAR assay data (Protocol 2) on heat-inactivated SARS-CoV-2 virus (Sample Batch 3, *n* = 3), showing (*A*) the mean time for the amplification reaction using RTF-EXPAR, (*B*) the mean time for the amplification reaction using RT-LAMP, and (*C*) the mean time for the amplification reaction using RT-qPCR. Each run time was calculated to be the point at which the fluorescence signal was greater than 10 SDs from the baseline signal (10-sigma time). Error bars in datasets are the SDs of the 10-sigma time.

Having established that the RTF-EXPAR assay could amplify isolated RNA faster than both RT-qPCR and RT-LAMP with comparable sensitivities, next we tested the three techniques on heat-inactivated SARS-CoV-2 virus (Sample Batch 3) using Protocol 2. Once again, a dilution study was performed, ranging from 4.2 × 10^5^ to 0.42 viral copies per µL (Fig. 4). As these samples had not been subjected to RNA extraction, we were anticipating lower sensitivities and longer reaction times for all three techniques. However, in terms of sensitivity, RT-qPCR was by far the most affected, for which only the most concentrated sample (4.2 × 10^5^ viral copies per µL) produced a signal change faster than that for the blank (43.67 ± 0.47 min compared to 49.67 ± 2.36 min, respectively). RT-LAMP was once again faster than RT-qPCR but also much more sensitive, with all samples at concentrations of 420 viral copies per µL producing a signal change before the nonspecific rise time of 35.00 ± 3.54 min. This improved sensitivity was mirrored for RTF-EXPAR, with its LOD (420 viral copies per µL) three orders of magnitude lower than RT-qPCR. The reaction time for the LOD concentration (7.67 ± 0.24 min) was over six times faster than the corresponding times for RT-qPCR (49.33 ± 1.25 min) and twice as fast as RT-LAMP (15.75 ± 0.20 min).

**Fig. 5.**
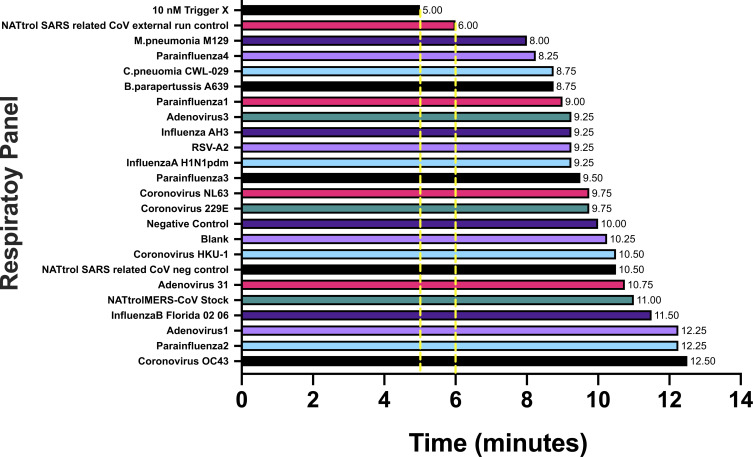
RTF-EXPAR assay data (Protocol 2) for ZeptoMetrix NATtrol Respiratory Verification Panel 2 (Sample Batch 4, *n* = 1), showing the time for RTF-EXPAR to produce a signal. Yellow dashed lines represent the thresholds for each of the two positive controls. Each run time was calculated to be the point at which the fluorescence signal was greater than 10 SDs from the baseline signal (10-sigma time). It should be noted that runs against *Influenza ah 1 a/newcal/20/99* and *Rhinovirus type 1a* gave no signal after 40 min.

Finally, we undertook specificity tests by comparing the ability of RTF-EXPAR to identify SARS-CoV-2 among a range of common respiratory pathogens using Protocol 2 (Sample Batch 4, all of which had been detected by their respective PCR assay on the same threshold cycle, C_T_ = 27). Both positive controls (one of which contained **Trigger X** only instead of **Binder DNA** **X** and SARS-CoV-2 RNA) were identified faster than 21 other pathogenic targets; these included four other coronaviruses as well as influenza and adenoviruses (Fig. 5).

In conclusion, through the use of an isothermal, reverse transcription-free (RTF) amplification method, RTF-EXPAR, involving a DNA-selective restriction endonuclease, we have demonstrated the successful detection of SARS-CoV-2 RNA in a total assay time of less than 10 min. In comparison to existing molecular tests, RTF-EXPAR holds a number of advantages. First, it is not only much faster than RT-qPCR but also outperforms the fastest nucleic acid testing method (RT-LAMP) that is currently in widespread use. This increase in speed has the potential to substantially increase the throughput of testing efforts without a concomitant increase in instrumentation time. Perhaps more importantly, the decrease in assay time to below 15 min makes the test more amenable for testing the public in primary-care settings. Second, the “one-pot” format of the RTF-EXPAR assay, combined with its speed, further increases the ease with which the test could be deployed away from clinical testing laboratories. Its community use could have significance for entertainment venues and border zones where testing has to be rapid but where the limitations of other rapid formats, like lateral flow, may prove problematic. Third, the use of trigger sequences in EXPAR that are shorter than those in both PCR and LAMP mean that the test is statistically less likely to be influenced by sample degradation. Finally, we should not lose sight of the wider application of an ultrafast RNA test with the sensitivity of PCR. Outside times of pandemic, testing for viral pathogens is a very important aspect of our efforts to control plant, animal, and human disease. In almost all of these cases the presence of a faster, simpler, and more easily deployable viral RNA test would provide a significant step change for molecular diagnostics.

## Materials and Methods

### Materials.

Milli-Q water purified with a Millipore Elix-Gradient A10 system (resistivity >18 μΩ⋅cm, total organic carbon ≤ 5 ppb) was used in all the experiments. Nt.*Bst*NBI, *Bst*NI, and *Bst* 2.0 Polymerase were obtained from New England Biolabs, as was the buffer, 10× isothermal amplification buffer [200 mM Tris⋅HCl, 100 mM (NH_4_)_2_SO_4_, 500 mM KCl, 20 mM MgSO_4_, and 1% Tween 20, pH 8.8] which was used in all the experiments. Superscript IV Reverse Transcriptase was obtained from Thermo Fisher, dimethyl sulfoxide (DMSO) (≥99%) was obtained from Fisher Scientific, and dsGreen 100× (an analog of SYBR Green I) was obtained from Lumiprobe. Bovine serum albumin (BSA, diluted to 4 mg/mL in water) and Single-Stranded Binding Protein (SSB, solution of 0.5 mg in 20 mM Tris⋅HCl, pH 8.0, 0.5 M NaCl, 0.1 mM ethylenediaminetetraacetic acid, 0.1 mM dithiothreitol, and 50% glycerol) was obtained from Sigma-Aldrich. All the nucleotide triphosphates and oligonucleotide sequences (desalted) were obtained from Sigma-Aldrich.

### Viral Samples.

All samples were handled in a Containment Level 2 laboratory.

#### Sample Batch 1.

A P2 stock of virus was acquired from High Containment Microbiology, PHE, Porton Down, corresponding to the SARS-CoV-2/human/AUS/VIC16832/2020 isolate, which was originally isolated in Australia from a COVID-19 patient in 2020. To prepare samples for RNA extraction, media containing the virus was added to Buffer AVL (Qiagen) in a 1/5 ratio and heated to 60 °C for 30 min in a calibrated heat block. Samples were then extracted on the MagNAPure96 (Roche) automated extraction system and then run on the Abbott M2000 RT-qPCR Test for SARS-CoV-2 RNA Detection. For EXPAR assay development, positive and negative RNA samples from the SARS-CoV-2 assays were separately combined in MagNA Pure elution buffer (giving 29,080 RNA copies per µL for the combined positive sample). Upon receipt from PHE, each sample (positive and negative) was diluted 400-fold with water, aliquoted into 50 µL vials, and stored at −80 °C.

#### Sample Batch 2.

The source of SARS-CoV-2 virus and RNA extraction procedure were the same as that described above for Sample Batch 1 except that the final RNA concentration was 29,000 copies per µL. The positive sample was serially diluted with water, with each diluted specimen then stored at −80 °C.

#### Sample Batch 3 (heat-inactivated virus).

ATCC VR-1986HK Heat-inactivated SARS-CoV-2 virus was serially diluted with water, with each diluted specimen then stored at −80 °C.

#### Sample Batch 4 (respiratory panel).

ZeptoMetrix NATtrol Respiratory Verification Panel 2 was used as supplied.

### RTF-EXPAR Assay Protocol 1.

The protocol first involves the preparation of three solutions, Part A, Part B, and Part C (each mixed in the reagent order given), followed by an addition step and then finally an amplification step. Prior to use, each frozen RNA sample was submerged in ice and allowed to slowly melt; once melted, the required amount of sample was used immediately before the remainder was frozen again for storage at −80 °C. Other biological reagents were slowly thawed on ice, with other reagents thawed at room temperature (21 °C).

#### Part A.

1.50 µL of water, 2.50 µL of 10× Isothermal amplification buffer, 3.75 µL of BSA solution, 1.50 µL of *Bst* 2.0 DNA polymerase (1.6 U/µL) and then 0.75 µL of Nt.*Bst*NBI (10 U/µL).

#### Part B.

6.30 µL of water, 5.00 µL of 10× Isothermal amplification buffer, 0.75 µL of **Template X′-X′** (1 µM), 2.40 µL of MgSO_4_ (100 mM), 1.50 µL dNTP (10 nM), 0.75 µL of dsGreen (1:5 dilution in DMSO from 100× to 20×) and then 0.30 µL of SSB solution.

#### Part C.

1) Sensitivity test (no RNA target): 3 µL of one trigger at **Trigger X** (100 nM, 10 nM, 1 nM, 100 pM, 10 pM, 1 pM, and a blank).

OR

2) Specificity test (no RNA target): 3 µL of one trigger at 100 nM (**Trigger X** or **Trigger A** or **Trigger B** or **Trigger C**).

OR

3) RTF EXPAR assay (two-pot RTF-EXPAR): 10 µL of RNA:DNA heteroduplex digestion mixture, prepared as follows: 25 µL of water, 5 µL of 10× Isothermal amplification buffer, 5 µL *Bst*NI (10 U/µL), 10 µL of **Binder DNA** **X** (1 µM), and then 5 µL of positive or negative sample (Sample Batch 1). The mixture is then incubated at 50 °C for 5 min.

OR

4) RTF EXPAR assay (one-pot RTF-EXPAR): Reagents are mixed together as follows: 1 µL *Bst*NI (10 U/µL), 2 µL of **Binder DNA** **X** (1 µM), and then 3 µL of positive or negative sample (Sample Batch 1).

##### Addition step.

Part B (17 µL) is added to a PCR tube, and to this is added Part C, followed by Part A (10 µL). The tube is then sealed and the contents subjected to amplification.

##### Amplification step.

Isothermal incubation and fluorescence signal measurements are performed using an Agilent Mx3005P Real-Time PCR system. The temperature is set at 25 °C for 15 s before being raised to 50 °C for the duration of the assay, with the fluorescence reading measured every 10 s over an incubation time of 30 min.

### RTF-EXPAR Assay Protocol 2.

This protocol was identical to the one-pot protocol described above with the exception that a 10-fold reduction in concentration of **Binder DNA** **X** in Part C, 4 was used (2 µL of a 100 nM solution), with isothermal incubation and fluorescence signal measurements performed using a Thermo Fisher QuantStudio 5 Real-Time PCR system, 96-well, 0.2 mL. All blank runs were run under identical conditions (reagents and volumes) but in the absence of the positive sample (i.e., 3 µL of sample in Part C, 4 contained water only).

### LAMP Protocol.

RTF-LAMP was performed using New England Biolabs WarmStart LAMP Kit with SARS-CoV-2 LAMP Primers in concordance with the manufacturer’s instructions. The equipment used was the same as that for Protocol 2.

### PCR Protocol.

RT-qPCR was performed using VIASURE Real Time PCR Detection kit in concordance with the manufacturer’s instructions. The equipment used was the same as that for Protocol 2.

### Data Analysis and Classification.

To analyze the EXPAR real-time fluorescence amplification curves and data, a program in C# was developed. The program analyses the first 10 data points and calculates the mean value and SD as a base line. Following generation of these two values, each subsequent data point is analyzed to determine if its value minus the average value is greater than 10 SDs away from the mean. The cycle which meets this criterion is converted into a time and used as the minimum amplification time.

## Supplementary Material

Supplementary File

## Data Availability

Primary fluorescence data and data analysis code for the determination of 10-sigma times, written in C#, can be obtained from the The Dryad Digital Repository: https://doi.org/10.5061/dryad.k0p2ngf8s (23).
