# Supplementary material for: Ultrarapid detection of SARS-CoV-2 RNA using a reverse transcription–free exponential amplification reaction, RTF-EXPAR
Source: Proc Natl Acad Sci U S A. 2021 Aug 16;118(35):e2100347118. doi: 10.1073/pnas.2100347118 (PMC8536344; doi:10.1073/pnas.2100347118)
Supplement: Supplementary File [file pnas.2100347118.sapp.pdf]

Ultra-rapid Detection of SARS-CoV-2 RNA using a Reverse Transcription-Free Exponential Amplification Reaction, RTF-EXPAR

Jake G. Carter,[1,2] Lorea Orueta Iturbe,[3] Jean-Louis H. A. Duprey,[3] Ian R. Carter,[4] Craig D. Southern,[4] Marium Rana,[1,2] Celina Whalley,[5] Andrew Bosworth,[5,6] Andrew D. Beggs,[5] Matthew R. Hicks,[3] James H. R. Tucker[1]* and Timothy R. Dafforn[2]*

1. School of Chemistry, University of Birmingham, Edgbaston, Birmingham, UK, B15 2TT.
2. School of Biosciences, University of Birmingham, Edgbaston, Birmingham, UK, B15 2TT.
3. Linear Diagnostics Ltd, 97 Vincent Drive, Edgbaston, Birmingham, UK, B15 2SQ.
4. Institute of Cancer and Genomics, University of Birmingham, Edgbaston, Birmingham, UK, B15 2TT.
5. C2JF Solutions LLP, 35 Meridian Business Village, Speke, Liverpool, UK, L24 9LG.
6. Clinical Virology, Clinical Laboratory Services, University Hospitals Birmingham NHS Foundation Trust, Birmingham, UK, B15 2GW.

Contents:

1. EXPAR Sensitivity and Specificity Tests — Page S2
2. RTF-EXPAR Assay Data — Page S4
3. Assay Design Considerations — Page S8

1. EXPAR Sensitivity and Specificity Tests

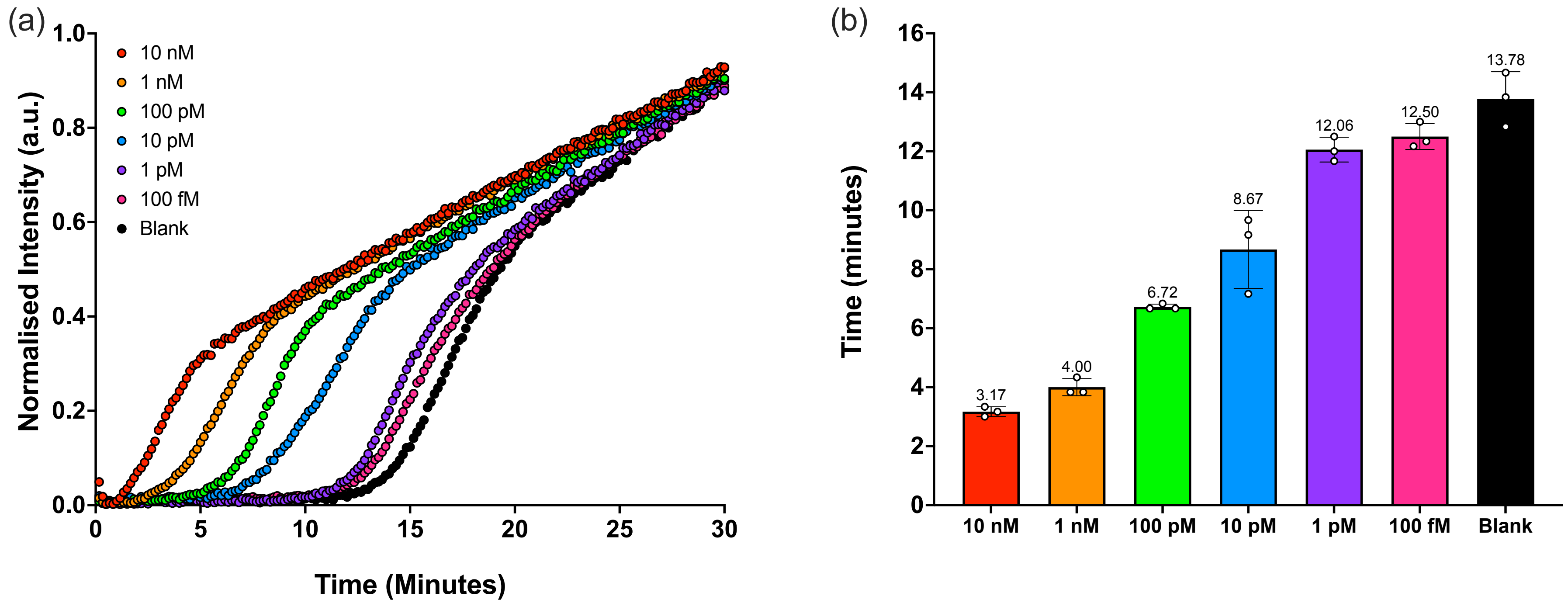

Figure S1. EXPAR sensitivity test data (Protocol 1) showing: (a) normalised average fluorescence intensity data (a.u) plotted against time (min) and (b) mean amplification times to greater than 10 standard deviations from the baseline fluorescence, plotted against **Trigger X** concentration (10 nM, 1 nM, 100 pM, 10 pM, 1 pM, 100 fM and a blank) in the presence of **Template X´-X´** (25 nM). Runs performed in triplicate (n = 3). Error bars in datasets are the standard deviations of the 10-sigma time. For other reagents and conditions, see RTF-EXPAR Protocol 1 (i).

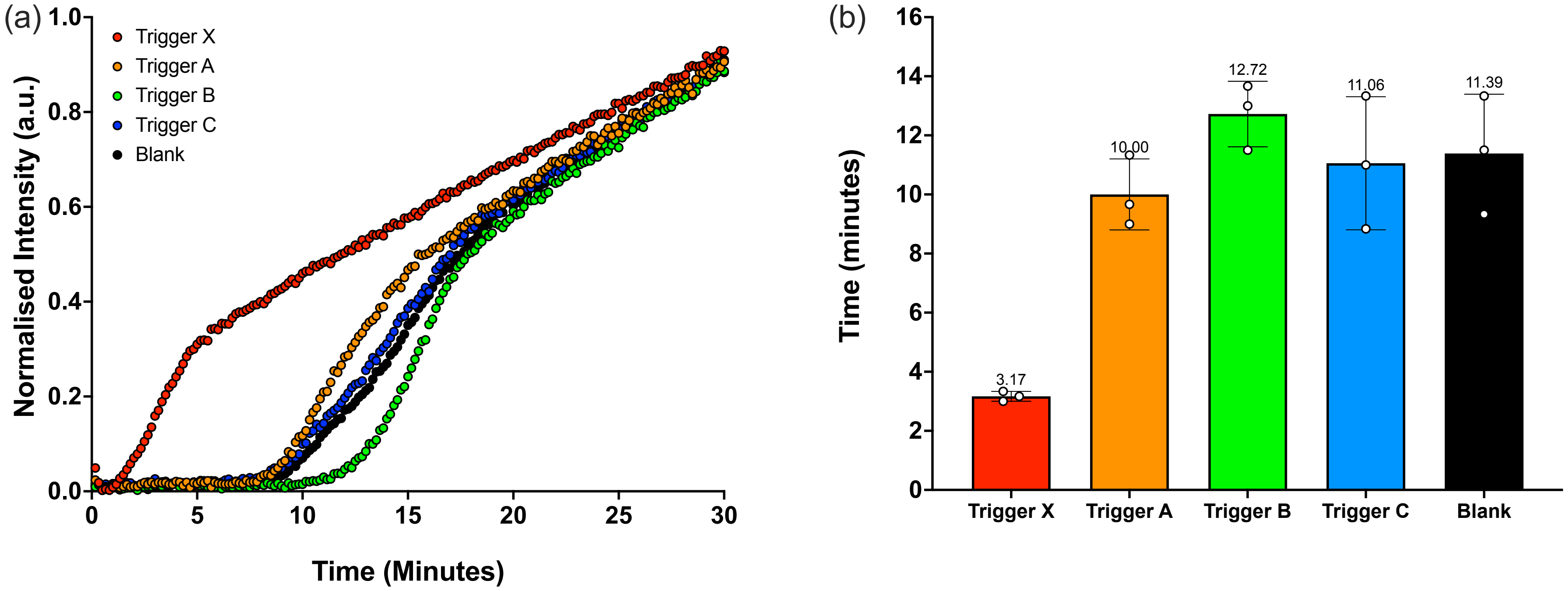

Figure S2. EXPAR specificity test data (Protocol 1) showing: (a) normalised average fluorescence intensity data (a.u) plotted against time (min) and (b) mean amplification times to greater than 10 standard deviations from the baseline fluorescence signal, plotted against **Trigger X** (fully complementary), **Trigger A** (non-complementary), **Trigger B** (non-complementary), **Trigger C** (non-complementary) and a blank (no trigger). **Template X´-X´** concentration 25 nM, trigger concentration 10 nM. Runs performed in triplicate (n = 3). Error bars in datasets are the standard deviations of the 10-sigma time. For other reagents and conditions, see RTF-EXPAR Protocol 1 (ii).

2. RTF-EXPAR Assay Data

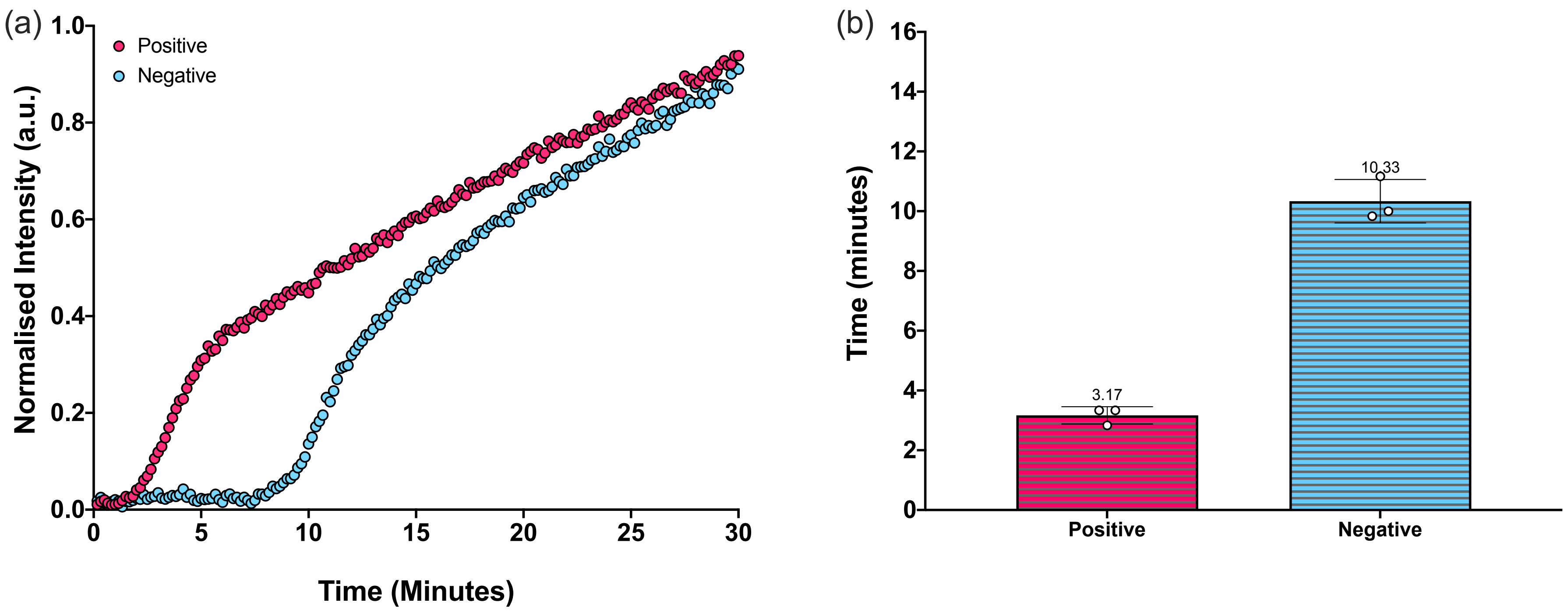

Figure S3. Two-Pot RTF-EXPAR data (Protocol 1) showing: (a) normalised average fluorescence intensity data (a.u) plotted against time (min) and (b) mean amplification times to greater than 10 standard deviations from the baseline fluorescence signal, plotted against positive SARS-CoV-2 RNA (73 copies/µL, Sample Batch 1) and a negative (no RNA). Runs performed in triplicate (n = 3). Error bars in datasets are the standard deviations of the 10-sigma time. For other reagents and conditions, see RTF-EXPAR Protocol 1 (iii).

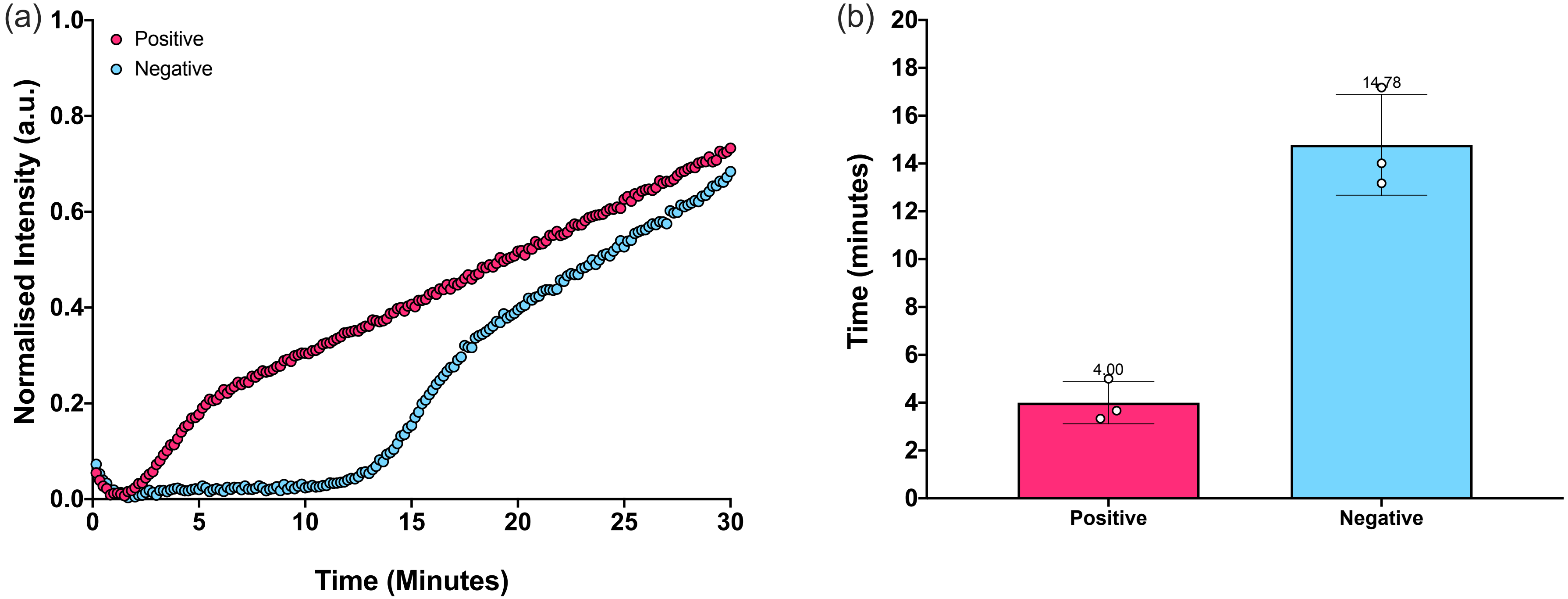

Figure S4. One-Pot RTF-EXPAR data (Protocol 1) showing: (a) normalised average fluorescence intensity data (a.u) plotted against time (min) and (b) mean amplification times to greater than 10 standard deviations from the baseline fluorescence signal, plotted against positive SARS-CoV-2 RNA (73 copies/µL, Sample Batch 1) and negative (no RNA). Runs performed in triplicate (n = 3). Error bars in datasets are the standard deviations of the 10-sigma time. For other reagents and conditions, see RTF-EXPAR Protocol 1 (iv).

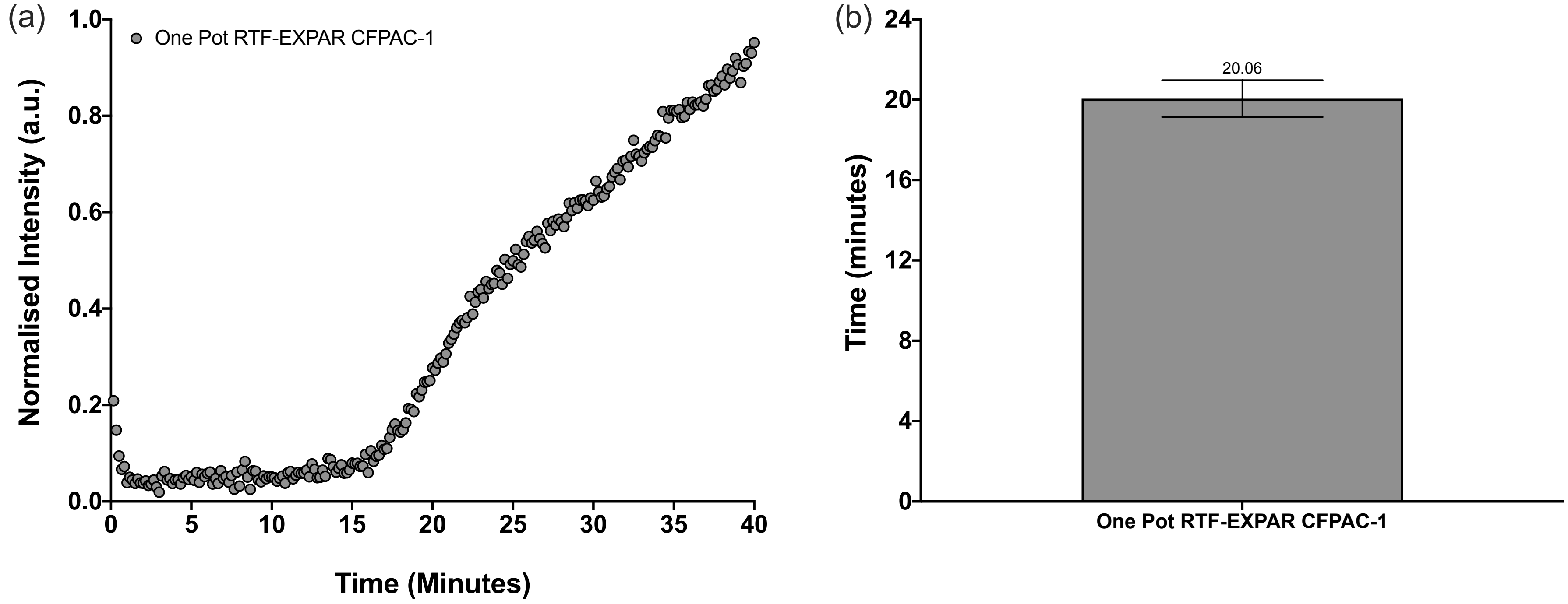

Figure S5. One-Pot RTF-EXPAR data (Protocol 1) showing: (a) normalised average fluorescence intensity data (a.u) plotted against time (min) and (b) mean amplification time to greater than 10 standard deviations from the baseline fluorescence signal, plotted against sample containing RNA isolated from the CFPAC-1 human ductal pancreatic adenocarcinoma cell line (160.3 ng/µL). Runs performed in triplicate (n = 3). Error bars in datasets are the standard deviations of the 10-sigma time. For other reagents and conditions, see RTF-EXPAR Protocol 1.

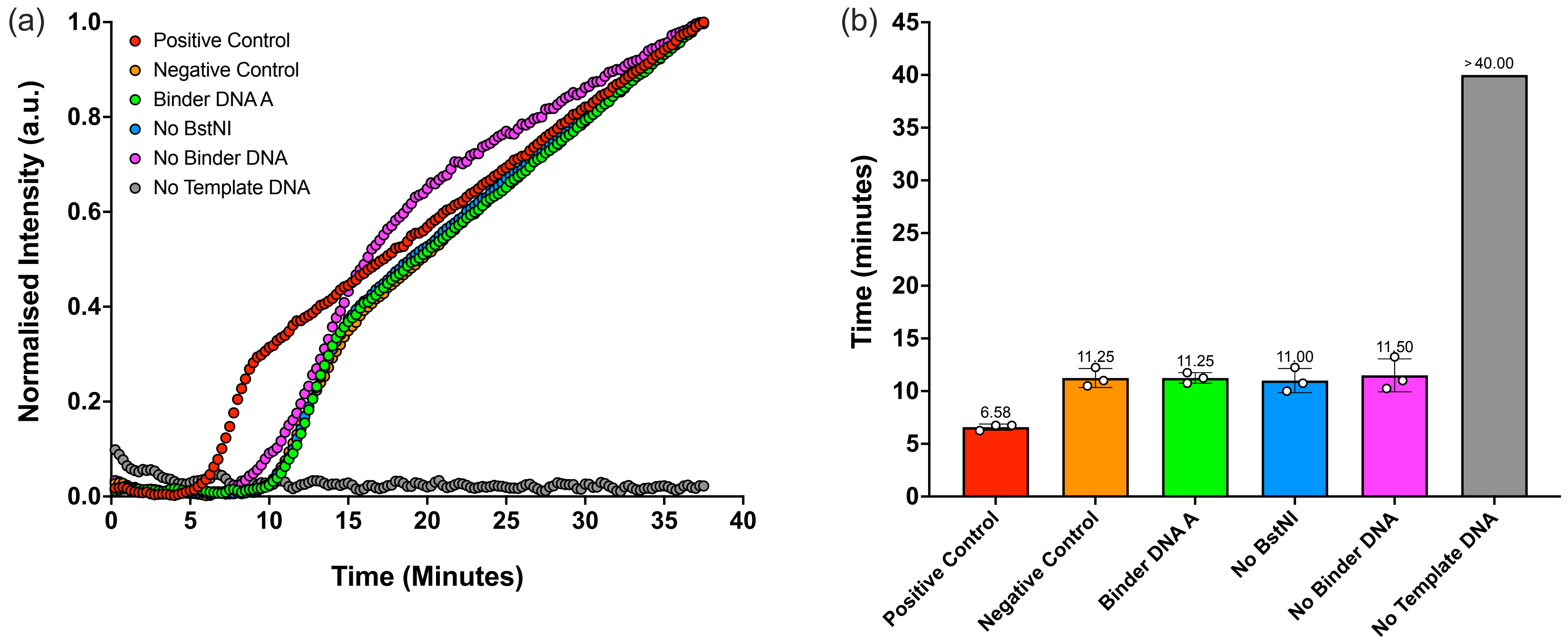

Figure S6. One-Pot RTF-EXPAR Control data (Protocol 2) showing: (a) normalised average fluorescence intensity data (a.u) plotted against time (min) and (b) mean amplification time to greater than 10 standard deviations from the baseline fluorescence signal. Data shows positive control (red, 73 copies/µL SARS-CoV-2 RNA) containing all components of RTF-EXPAR assay, and five negative controls containing all components of RTF-EXPAR but in the absence of: RNA (orange), **Binder DNA X**, (replaced by an alternative, **Binder DNA A,** green), *Bst*NI (blue), **Binder DNA** (pink) and **Template X′-X′** (grey). Runs performed in triplicate (n = 3). Error bars in datasets are the standard deviations of the 10-sigma time. For other reagents and conditions, see RTF-EXPAR Protocol 2. RNA used was Sample Batch 2.

3. Assay Design Considerations

The recognition site of *Bst*NI is 5′- CCWGG - 3′ (where W = any base**)**. Therefore we examined the cDNA sequence of the conserved gene *Orf1ab* from SARS-CoV-2 to find every restriction site (https://www.ncbi.nlm.nih.gov/nuccore/MN908947.3?report=fasta), of which we found ten. Out of the ten potential sequences to which a **Binder DNA** sequence could bind, four were immediately discarded as three possessed multiple restriction sites in close proximity to one another, and one other possessed a poly A region, which was deemed too unstable a binding region for the assay. Next, we added the base sequence AGGGT to the 5′-end of the trigger component within the **Binder DNA**, with this sequence serving two roles:

1) To improve the efficiency of the EXPAR reaction, given that Qian *et al* had previously demonstrated the importance of having particular trigger base sequences adjacent to the Nt.*Bst*NBI nicking site. (1, 2)
2) To act as both a non-RNA-binding region in the **Binder DNA** sequence and a DNA-binding region in the trigger sequence, which would sufficiently raise the T_m of the trigger to allow it to bind to the template, once it had been cleaved from the **Binder DNA**.

Following the inclusion of these bases we performed *in silico* analysis of the melting temperatures of six potential **Binder DNA** and trigger strands with their respective complementary sequences. EXPAR is an isothermal amplification technique, but the three enzymes employed in the assay all function at different optimal temperatures. In order to appease the enzymes, we opted to run the reaction at 50 °C and assessed each trigger sequence to determine which would be on the cusp of its T_m at this reaction temperature. Those sequences with melting temperature values closest to 50 °C would be capable of both binding to the template, thus initiating EXPAR, yet also being able to be released upon nicking. Assessment of the six remaining sequences yielded three sequences that could potentially fit this criteria, **Trigger A**, **Trigger B** and **Trigger X** (Figure 1a and Table 1). Following initial EXPAR trials we determined that **Trigger X** produced the largest separation between positive and non-specific amplification and was therefore carried forward.

1. J. Qian, *et al.*, Sequence dependence of isothermal DNA amplification via EXPAR. *Nucleic Acids Research* **40**, e87–e87 (2012).
2. M. S. Reid, R. E. Paliwoda, H. Zhang, X. C. Le, Reduction of Background Generated from Template-Template Hybridizations in the Exponential Amplification Reaction. *Anal. Chem.* **90**, 11033–11039 (2018).
